# Inhibition of ANO1/TMEM16A Chloride Channel by Idebenone and Its Cytotoxicity to Cancer Cell Lines

**DOI:** 10.1371/journal.pone.0133656

**Published:** 2015-07-21

**Authors:** Yohan Seo, Jinhong Park, Minseo Kim, Ho K. Lee, Jin-Hee Kim, Jin-Hyun Jeong, Wan Namkung

**Affiliations:** 1 Department of Integrated OMICS for Biomedical Science, Graduate School, Yonsei University, Seoul, 120–749, Korea; 2 College of Pharmacy, Yonsei Institute of Pharmaceutical Sciences, Yonsei University, Incheon, 406–840, Korea; Monell Chemical Senses Center, UNITED STATES

## Abstract

The expression levels of anoctamin 1 (ANO1, TMEM16A), a calcium-activated chloride channel (CaCC), are significantly increased in several tumors, and inhibition of ANO1 is known to reduce cell proliferation and migration. Here, we performed cell-based screening of a collection of natural products and drug-like compounds to identify inhibitors of ANO1. As a result of the screening, idebenone, miconazole and plumbagin were identified as novel ANO1 inhibitors. Electrophysiological studies showed that idebenone, a synthetic analog of coenzyme Q10, completely blocked ANO1 activity in FRT cells expressing ANO1 without any effect on intracellular calcium signaling and CFTR, a cAMP-regulated chloride channel. The CaCC activities in PC-3 and CFPAC-1 cells expressing abundant endogenous ANO1 were strongly blocked by idebenone. Idebenone inhibited cell proliferation and induced apoptosis in PC-3 and CFPAC-1 cells, but not in A549 cells, which do not express ANO1. These data suggest that idebenone, a novel ANO1 inhibitor, has potential for use in cancer therapy.

## Introduction

Calcium-activated Cl^-^ channels (CaCCs) are widely expressed in various cell types and tissues, and they are implicated in many physiological activities such as epithelial fluid secretion, smooth muscle contraction, and sensory signal transduction [1–3]. CaCCs were first described over 3 decades ago but the molecular identity of CaCCs has recently been identified [4]. In 2008, three independent research groups reported that anoctamin-1 (ANO1, TMEM16A) gene encodes a CaCC, showing calcium-activated Cl^-^ currents when it was expressed in oocytes and mammalian cells [5–7]. ANO1 is expressed in various cell types including tracheal, intestinal, and glandular epithelia, smooth muscle cells, intestinal pacemaker cells, sensory neurons, and several tumors [5, 7–9].

ANO1 was also known as discovered on GIST-1 (DOG1), tumor amplified and overexpressed sequence 2 (TAOS2), and oral cancer overexpressed 2 (ORAOV2) [10, 11]. DOG1, TAOS2 and ORAOV2 are named so because ANO1 is strongly overexpressed in gastrointestinal stromal tumours (GIST) and oral squamous cell carcinomas. ANO1 is mapped to the chromosomal band 11q13 that is frequently amplified in a variety of human carcinomas including head-and-neck squamous cell carcinoma (HNSCC), GIST, breast and prostate cancer. Recent evidence suggests ANO1 involvement in cell proliferation, cell migration, tumorigenesis and cancer progression [12, 13]. For instance, inhibition of ANO1 expression in prostate cancer PC-3 cells significantly reduced proliferation, metastasis and invasion, and blocked tumor growth in a xenograft mouse model [14]. Pharmacological inhibition of ANO1 by T16A_inh_-A01, a selective ANO1 inhibitor, reduced proliferation of interstitial cells of Cajal (ICC) and CFPAC-1 pancreatic cancer cells expressing endogenous ANO1 [15]. In breast cancer cells, down-regulation of the ANO1 gene expression reduced proliferation, provoked apoptosis, and inhibited tumor growth in a xenograft model. In addition, pharmacological inhibition of CaCC activity of ANO1 reduced cell viability in HNSCC, esophageal squamous cell carcinoma (ESCC) and breast cancer cells via inhibition of epidermal growth factor receptor (EGFR) and calmodulin-dependent protein kinase II (CAMKII) signaling [16].

Most evidence indicates that pharmacological inhibition of ANO1 channel activity may have the potential to provide therapeutic benefits to HNSCC, ESCC, GIST, breast and prostate cancer patients. Since ANO1 has recently been identified, only few compounds were identified as potent ANO1 inhibitors such as CaCC_inh_-A01, tannic acid, T16A_inh_-A01, digallic acid, dichlorophen, benzbromarone, and N-((4-methoxy)-2-naphthyl)-5-nitroanthranilic acid (MONNA). Moreover, pharmacological property and the mechanisms of action of the inhibitors are still unclear [17–21].

For the identification of novel ANO1 inhibitors, we performed a cell-based screening with a collection of natural products and drug-like compounds using a cell based high-throughput screening assay established for the identification of ANO1 inhibitors in previous study [19]. We found some drug-like compounds and natural products showing potent ANO1 inhibitory activity, and investigated the effect of the hit compounds on growth inhibition of cancer cell lines, which express ANO1 endogenously.

## Materials and Methods

### Materials and solutions

Idebenone, coenzyme Q10, plumbagin, miconazole, and other chemicals, unless otherwise indicated, were purchased from Sigma. Mouse ANO2 was purchased from Origene Technologies Inc. (Rockville, MD, USA, catalog No MC205812). The compound collection used for screening (Spectrum Collection, 2320 compounds) was purchased from MicroSource Discovery Inc. (Gaylordsville, CT). This library consists of human therapeutic drugs or drug-like compounds and natural products. The compounds were diluted with DMSO to reach a concentration of 2.5 mM. This was used as the 100x concentrated stock solution which was treated on the cells.

### Cell culture

Fisher rat thyroid (FRT) cells were stably transfected with human ANO1(abc) or human wild-type CFTR separately, and both of the cells were stably transfected with the halide sensor YFP-H148Q/I152L/F46L or YFP-H148Q as described in previous study [20, 22]. FRT cells were stably transfected with mouse ANO2 (Origene Technologies Inc.) and the halide sensor YFP-F46L/H148Q/I152L. FRT cells cultured in Coon`s modified F12 medium supplemented with 10% FBS, 2 mM L-glutamine, 100 U/mL penicillin and 100 μg/mL streptomycin. PC3 and HT-29 cells were grown in RPMI 1640 medium supplemented with 10% FBS, 100 units/ml penicillin and 100 μg/ml streptomycin. A549 cells were cultured in Dulbecco's Modified Eagle Medium (DMEM) containing 10% FBS, 100 units/ml penicillin and 100 μg/ml streptomycin. CFPAC-1 cells were grown in Iscove's Modified Dulbecco's Medium (IMDM) supplemented with 10% FBS, 100 units/ml penicillin and 100 μg/ml streptomycin.

### Cell based screening

ANO1/TMEM16A and YFP expressing FRT cells were plated in 96-well black-walled microplates (Corning Inc., Corning, NY) at a density of 20,000 cells per well in F12 medium supplemented with 10% FBS, 100 U/mL penicillin, 100 μg/mL streptomycin. Assays were done using FLUOstar Omega microplate reader (BMG Labtech, Ortenberg, Germany) and MARS Data Analysis Software (BMG Labtech). Each well of 96-well plate was washed 3 times in PBS (200 μL/wash), leaving 100 μL PBS. Test compounds (1 μL) were added to each well at 25 μM final concentration. After 10 min, 96-well plates were transferred to a plate reader for fluorescence assay. Each well was assayed individually for TMEM16A-mediated I^-^ influx by recording fluorescence continuously (400 ms per point) for 2 s (baseline), then 100 μL of 140 mM I^-^ solution containing 200 μM ATP was added at 2 s and then YFP fluorescence was recorded for 6 s. Initial iodide influx rate was determined from the initial slope of fluorescence decrease, by nonlinear regression, following infusion of iodide with ATP.

### Ussing Chamber study

Snapwell inserts containing ANO1- or CFTR-expressing FRT cells were mounted in Ussing chambers (Physiologic Instruments, San Diego, CA). The basolateral bath was filled with HCO_3_^-^-buffered solution containing (in mM): 120 NaCl, 5 KCl, 1 MgCl_2_, 1 CaCl_2_, 10 D-glucose, 2.5 HEPES, and 25 NaHCO_3_ (pH 7.4), and the apical bath was filled with a half-Cl^-^ solution. In the half-Cl^-^ solution 65 mM NaCl in the HCO_3_^-^-buffered solution was replaced by Na-gluconate. The basolateral membrane was permeabilized with 250 μg/mL amphotericin B. Cells were bathed for a 20 min stabilization period and aerated with 95% O_2_ / 5% CO_2_ at 37°C. ATP was applied to the apical bath solution to induce intracellular calcium increase; idebenone, forskolin and CFTR_inh_-172 were added to the apical and basolateral bath solution. Apical membrane currents were measured with an EVC4000 Multi-Channel V/I Clamp (World Precision Instruments, Sarasota, FL) and recorded using PowerLab 4/35 (AD Instruments, Castle Hill, Australia). Data were collected and analyzed with ADInstruments acquisition software Labchart Pro 7 software. The sampling rate was 4 Hz.

### Patch-clamp

Whole-cell patch-clamp recordings were performed on ANO1-expressing FRT cells and PC3 cells. The bath solution contained (in mM): 140 NMDG-Cl, 1 CaCl_2_, 1 MgCl_2_, 10 glucose and 10 HEPES (pH 7.4). The pipette solution contained (in mM): 130 CsCl, 0.5 EGTA, 1 MgCl_2_, 1 Tris-ATP, and 10 HEPES (pH 7.2). Pipettes were pulled from borosilicate glass and had resistances of 3–5 MΩ after fire polishing. After establishing the whole-cell configuration, ANO1 was activated by ATP (100 μM). Whole-cell currents were elicited by applying hyperpolarizing and depolarizing voltage pulses from a holding potential of 0 mV to potentials between -80 mV and +80 mV in steps of 20 mV. Recordings were made at room temperature using an Axopatch-200B (Axon Instruments, Union City, CA). Currents were digitized and analyzed using a Digidata 1440A converter (Axon Instruments), and pCLAMP 10.2 software (Molecular Devices, Sunnyvale, CA). Currents were low-pass filtered at 1 kHz and sampled at 5 kHz.

### Immunoblot

Cell extracts and immunoblotting were prepared as described previously [23]. FRT-ANO1, PC3, CFPAC-1 and A549 cells were lysed with cell lysis buffer (50 mM Tris-HCl, pH 7.4, 1% Nonidet P-40, 0.25% sodium deoxycholate, 150 mM NaCl, 1 mM EDTA, 1 mM Na_3_VO_4_, and protease inhibitor mixture). Whole cell lysates were centrifuged at 15,000 g for 10 min at 4°C to remove the cell debris, and equal amounts (80 μg protein/lane) of supernatant protein were separated by 4–12% Tris-glycine precast gel (KOMA BIOTECH, Seoul, Korea) and then transferred onto PVDF membrane (Millipore, Billerica, MA). Membrane was blocked with 5% non-fat skim milk in Tris-buffered saline (50 mM Tris-Cl, pH 7.5, 150 mM NaCl) including 0.1% Tween 20 for 1 hour at room temperature. This membrane was then incubated overnight with primary ANO1 antibody (a generous gift of Young Duk Yang, CHA University). After washing with 0.1% Tween 20 in Tris buffered saline (TBST), the blot was further incubated for 45 min at room temperature with an anti-rabbit secondary antibody (Cell Signaling). The membrane was then washed three times with TBST for 5 minutes and then visualized using the ECL Plus western blotting detection system (GE Healthcare Amersham; Piscataway, NJ).

### Intracellular calcium measurement

FRT and HT-29 cells were cultured in 96-well black-walled microplates and loaded with Fluo-4 NW per the manufacturer's protocol (Invitrogen, Carlsbad, CA). Briefly, the cells were incubated with 100 μL assay buffer (1X Hanks’ balanced salt solution with 2.5 mM probenecid and 20 mM HEPES) including Fluo-4 NW. After 1 hour incubation, the 96 well plates were transferred to a plate reader for fluorescence assay. Fluo-4 fluorescence was measured with a FLUOstar Omega microplate reader (BMG Labtech) equipped with syringe pumps and custom Fluo-4 excitation / emission filters (485 / 538 nm). Intracellular calcium was increase by application of 100 μM ATP.

### Cell proliferation assays

PC3, CFPAC-1 and A549 cells were plated on 96-well microplates. After 24 hour incubation, cells were treated with different concentrations of idebenone (3, 10, 30 μM), coenzyme Q10 (100 μM) and T16A_inh_-A01 (10 μM), and then they were incubated for 2 days. An equal amount of DMSO was added to the all control. The culture medium and the compounds were changed every 12 h. To assess cell proliferation, after 48 hour incubation with the compounds, BrdU (final concentration: 10 μM) was added and the cells were reincubated for additional 2 hours. BrdU incorporation was determined by Cell Proliferation ELISA, BrdU (colorimetric) kit (Roche Applied Science, Indianapolis, IN). For MTS assay, after 48 hour incubation, the cells were reincubated with MTS for 1 hour. The soluble formazan produced by cellular reduction of MTS was quantified by measuring the absorbance at 490 nm with Infinite M200 (Tecan, Grödig, Austria) microplate reader. MTS assay was done using CellTiter 96 AQueous One Solution Cell Proliferation Assay kit (Promega, Madison, WI, USA).

### Wound healing assay

Cell mobility was assessed using a scratch wound assay. PC3 cells were cultured in a 96-well plate until it was confluent. The cell layer was wounded using a 96-Well WoundMaker (Essen BioScience, Michigan, USA) and washed twice with fresh serum free media. The cells were incubated with serum free medium, and images of the wounds were automatically taken every 2 h for 48 h using the IncuCyte ZOOM (Essen BioScience). The images were analyzed by the IncuCyte software package (Essen BioScience).

### TUNEL assays

PC3, CFPAC-1 and A549 cells were plated on 96-well black-walled microplates. After 24 hour incubation, cells were treated with idebenone (30 μM) and then incubated for 2 days. The cells were fixed and stained with TUNEL (terminal deoxynucleotide transferase-mediated dUTP nick-end labeling, green) using the ApopTag Fluorescein Direct In Situ Apoptosis Detection Kit (Millipore, Billerica, MA, USA), and then nucleus were stained with DAPI (4,6-diamidino-2-phenylindole, blue).

### Statistical analysis

The results of multiple experiments are presented as the means ± S.E. Statistical analysis was performed with Student’s t-test or by analysis of variance as appropriate. A value of *P* < 0.05 was considered statistically significant.

## Results

### Identification of ANO1/TMEM16A inhibitors

**Fig 1:**
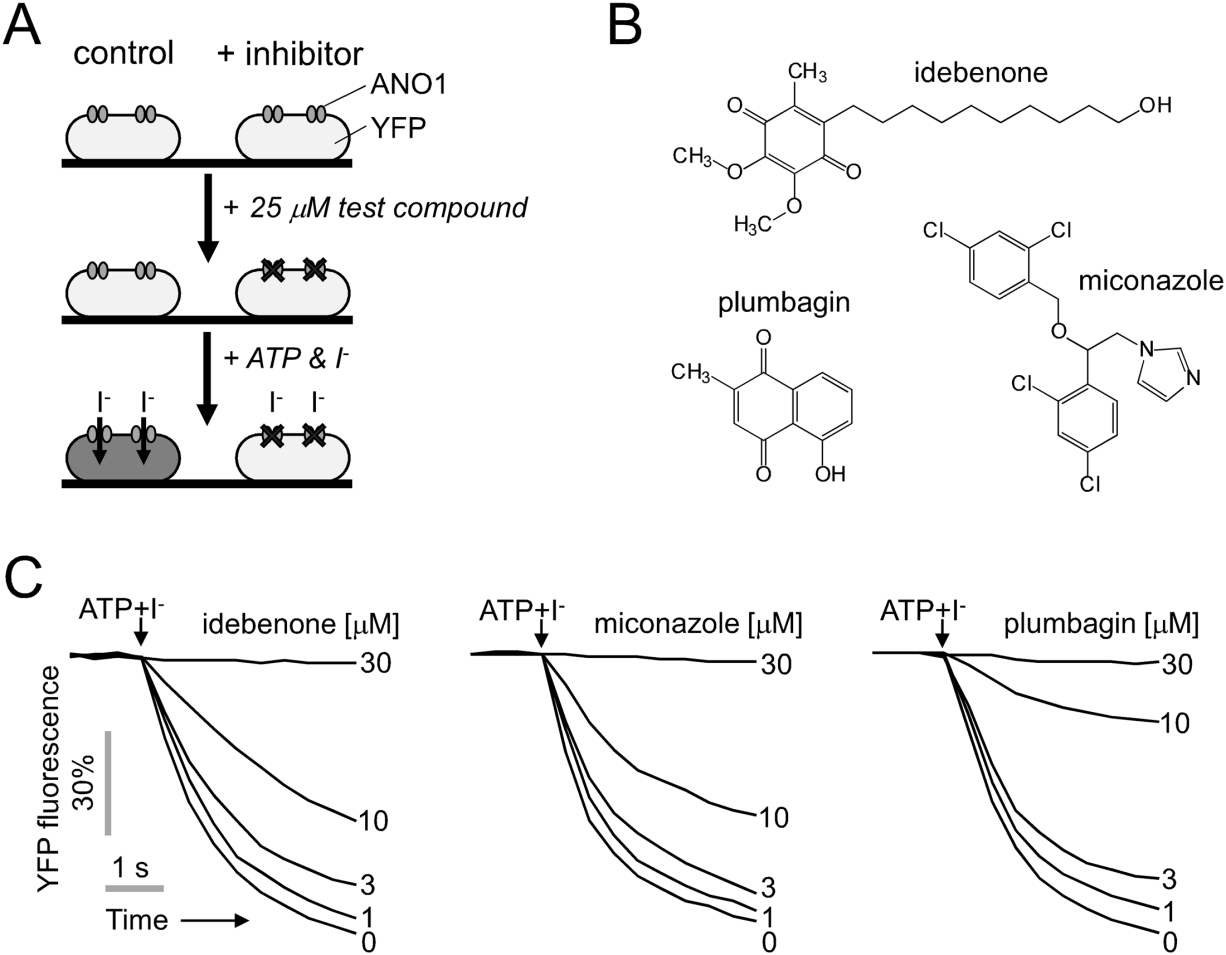
Identification of inhibitors of TMEM16A. (A) Principle of cell-based, fluorescence high-throughput screening assay. (B) Chemical structures of ANO1 inhibitors. (C) YFP fluorescence measured in single wells of 96-well plates, showing inhibitory effect of idebenone, miconazole and plumbagin on ANO1 channel activity. Indicated concentrations of idebenone, miconazole and plumbagin were added 20min prior to ANO1 activation by 100 μM ATP.

A cell-based screening of a collection of natural products and drug-like compounds was done for the identification of ANO1 inhibitors. ANO1 chloride channel activity was measured using Fischer rat thyroid (FRT) cells stably expressing human ANO1 and the genetically encoded iodide-sensing fluorescent protein, YFP-F46L/H148Q/I152L. As shown in Fig 1A, for the screening to identify ANO1 inhibitors, the FRT cells were pre-incubated with test compounds in PBS prior to addition of an iodide and ATP, an agonist of P2 purinergic receptor which causes an increase in intracellular calcium concentration, containing solution. In the presence of ANO1 inhibitor, YFP fluorescence quenching by iodide intake through ANO1 will be inhibited.

Screening of 2320 compounds yielded 24 compounds that blocked iodide influx by > 70% at 25 μM. We found three novel ANO inhibitors, idebenone, plumbagin and miconazole, from the primary hit compounds. Idebenone, plumbagin and miconazole significantly inhibited ANO1 chloride channel activity in a dose-dependent manner and fully inhibited ANO1 at 30 μM (Fig 1C).

### Characterization of idebenone

**Fig 2:**
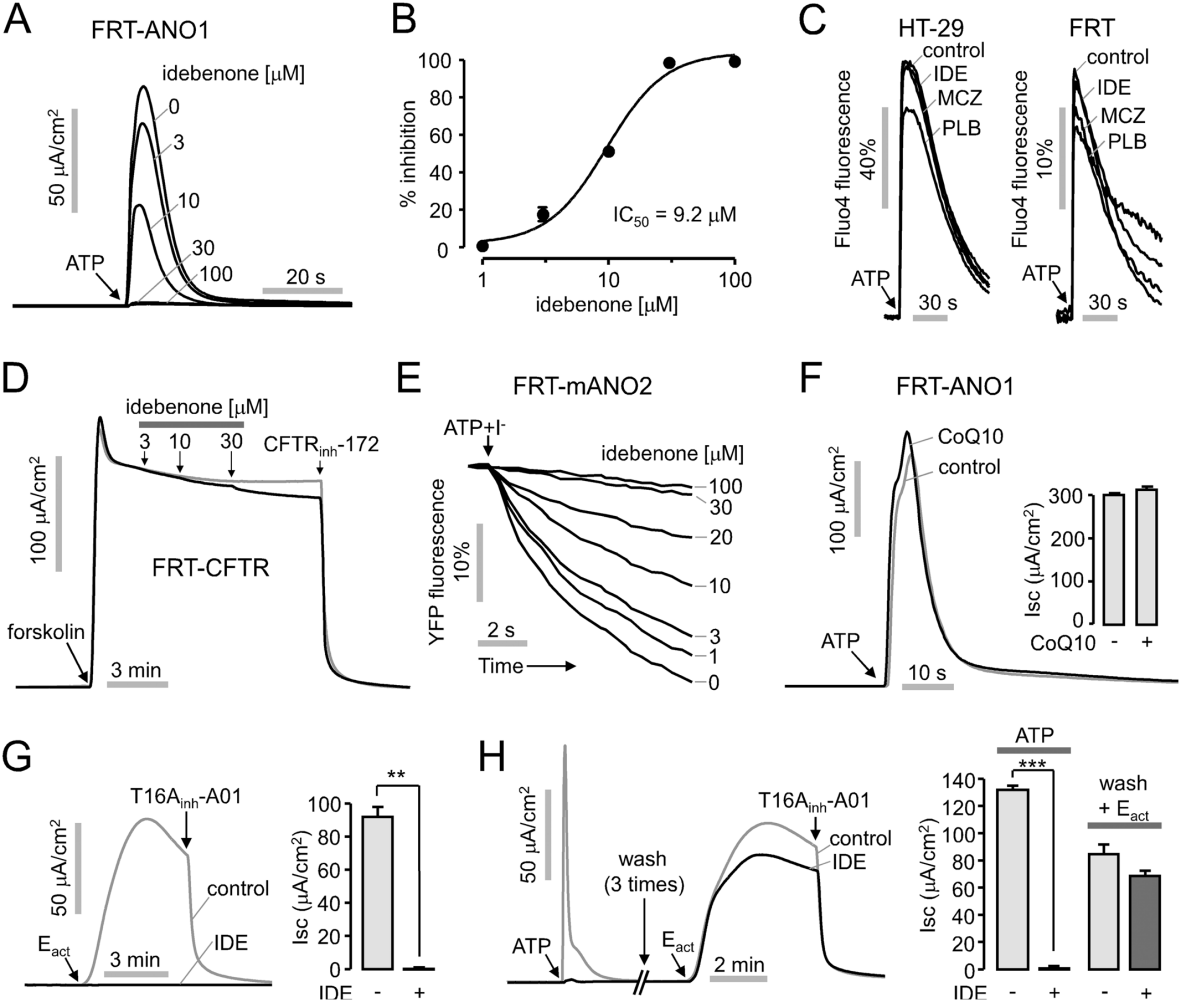
Characterization of idebenone, a TMEM16A inhibitor. (A) Apical membrane currents were measured in FRT-ANO1 cells. Idebenone was added 10 min prior to ANO1 activation by 100 μM ATP. (B) Summary of dose-response (mean ± S.E., n = 3–4). (C) Intracellular calcium concentration was measured using Fluo-4 in HT-29 and FRT cells. 30 μM idebenone (IDE), miconazole (MCZ) and plumbagin (PLB) were pretreated for 20min and then 100 μM ATP was applied. (D) Effect of idebenone on CFTR chloride channel activity was measured in FRT cells expressing human wild-type CFTR. CFTR was activated by 20 μM forskolin and inhibited by 10 μM CFTR_inh_-172. (E) Effect of idebenone on mouse ANO2 (mANO2) was measured in FRT-mANO2 cells. (F) Effect of Coenzyme Q10 (CoQ10) on ANO1 channel activity was observed in FRT-ANO1 cells. 100 μM CoQ10 was pretreated for 20min and then 100 μM ATP was applied. (right) Summary of peak current (mean ± S.E., n = 3–4). (G) Effect of idebenone on ANO1 activation by E_act_ in ANO1-expressing FRT cells. 100 μM idebenone (gray line) was pretreated for 20min and ANO1 was activated by 10 μM E_act_. The remaining ANO1 currents were inhibited by T16A_inh_-A01. (right) Summary of peak current (mean ± S.E., n = 3). (H) Idebenone reversibility. After vanishment of 100 μM ATP-induced ANO1 current, the cells were washed three times for 5 min each and then ANO1 was activated by 10 μM E_act_. (right) Summary of peak current (mean ± S.E., n = 3). **P < 0.01, ***P < 0.001, Students’ unpaired t-test.

Idebenone was further studied because idebenone, a synthetic analog of coenzyme Q10 (CoQ10), shows potent and selective inhibition of ANO1, and is widely used as a powerful antioxidant. Apical membrane currents measurement in ANO1-expressing FRT cells gave an IC_50_ of 9.2 μM for idebenone and show almost complete inhibition of ANO1 chloride currents by 30 μM idebenone (Fig 2A and 2B). To investigate the effect of idebenone on intracellular calcium signaling, HT-29 and FRT cells were loaded with Fluo-4 NW, a fluorescent calcium sensor. The cells were pretreated with 30 μM idebenone and then ATP applied at a concentration of 100 μM induced transient increase in the cytosolic calcium concentration. The ATP-induced cytosolic calcium increase was not significantly affected by idebenone (Fig 2C). To elucidate if idebenone alters the other chloride channels, cystic fibrosis transmembrane conductance regulator (CFTR) and ANO2 (TMEM16B), which shares a high amino acid homology to ANO1, we measured apical membrane currents in FRT cells expressing human wild-type CFTR and mouse ANO2 (mANO2). CFTR channel activity was affected only a little by idebenone. It inhibited CFTR by 8.2 ± 0.2% at 30 μM, a concentration that fully inhibits ANO1 (Fig 2D), but, not surprisingly, idebenone strongly inhibited 100 μM ATP-induced activation of mANO2 in a dose dependent manner in FTR-mANO2 cells (Fig 2E). We measured apical membrane currents to observe the effect of CoQ10 on ANO1 channel activity in FTR-ANO1 cells because idebenone is a short-chain analog of CoQ10. As shown in Fig 2F, 100 μM CoQ10 did not inhibit ATP-induced ANO1 chloride currents. To investigate whether idebenone reversibly inhibits ANO1, we measured apical membrane currents in FTR-ANO1 cell with E_act_, a specific activator of ANO1. Pretreatment of 100 μM idebenone completely inhibited E_act_-induced ANO1 activation (Fig 2G). As shown in Fig 2H, pretreatment of 100 μM idebenone completely inhibited ATP-induced ANO1 activation. However after washout, most of the inhibitory effect of idebenone on ANO1 activation by E_act_ was removed. Idebenone thus reversibly inhibits ANO1 without alterations in intracellular calcium signaling.

### Whole-cell patch clamp in FRT-ANO1 and PC-3 cells

**Fig 3:**
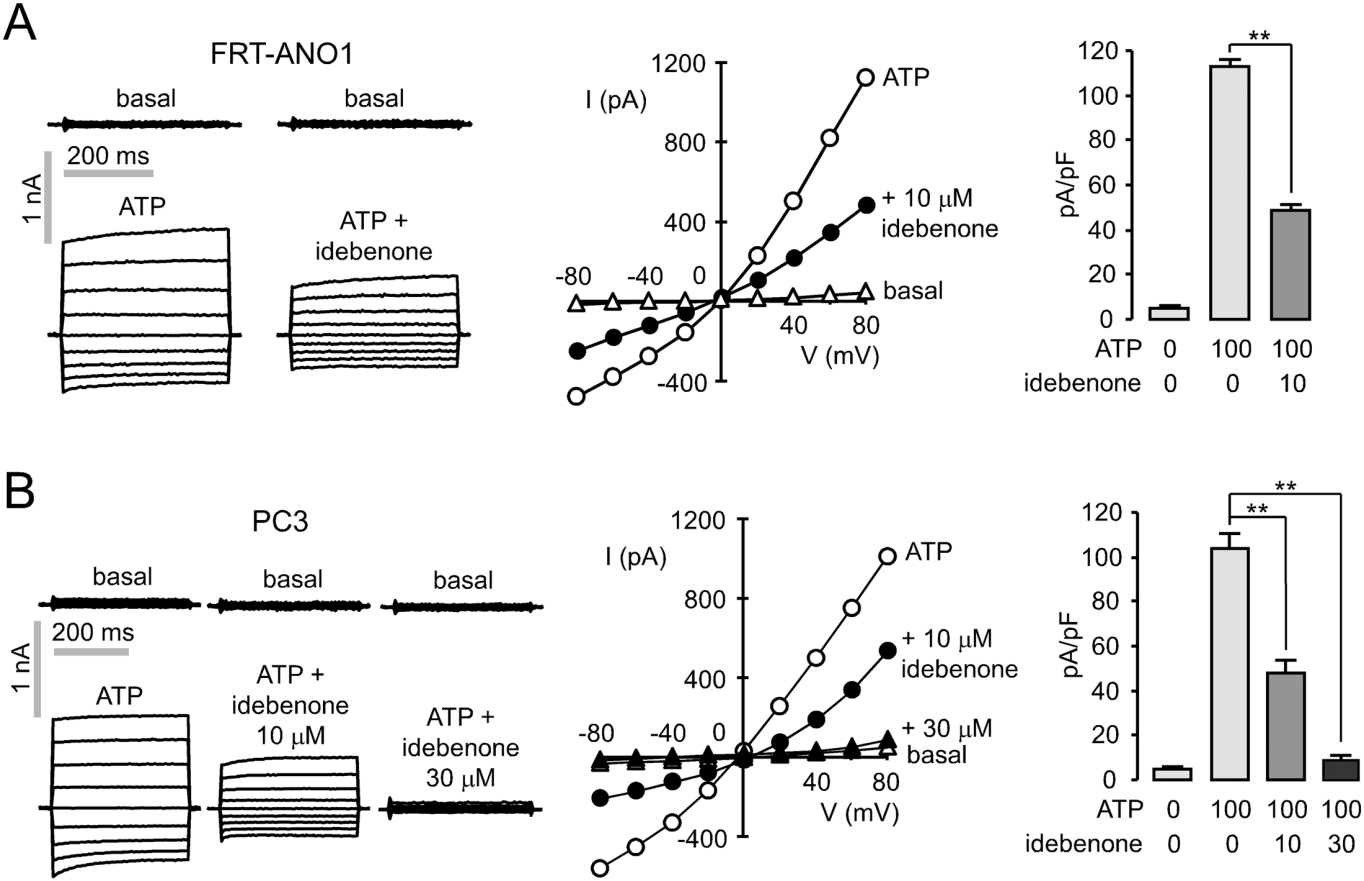
Idebenone inhibits ANO1 in FRT-ANO1 and PC3 cells. (A) (left) Whole-cell ANO1 current recorded at a holding potential at 0 mV and pulsing to voltages between ± 80 mV (in steps of 20 mV) in the absence and presence of 10 μM idebenone. ANO1 was activated by 100 μM ATP. (center) Current/voltage (I/V) plot of mean currents at the middle of each voltage pulse. (right) The bar graphs summarize current density data measured at + 80 mV (mean ± S.E., n = 4). (B) (left) Whole-cell patch clamp recordings of PC3 cells. CaCC currents recorded in the absence and presence of idebenone. CaCC was stimulated by 100 μM ATP. (center) Current/voltage (I/V) plot of mean currents at the middle of each voltage pulse. (right) The bar graphs summarize current density data measured at + 80 mV (mean ± S.E., n = 4). **P < 0.01, Students’ unpaired t-test.

In Fig 3, whole-cell patch clamp analysis was done to determine inhibition mechanisms of idebenone. In FRT cells expressing ANO1, application of 10 μM idebenone inhibited ATP-induced ANO1 chloride currents at all voltages, indicating that it has the inhibitory effect through a voltage-independent mechanism (Fig 3A). Patch clamp measurement of whole-cell currents in PC-3 (human prostate adenocarcinoma derived) cells expressing high level of ANO1 showed that idebenone at 10 μM and 30 μM inhibits ATP-induced CaCCs chloride currents by ~54% and ~90%, respectively (Fig 3B).

### ANO1 inhibitors decrease cell proliferation and migration in adenocarcinoma cells

**Fig 4:**
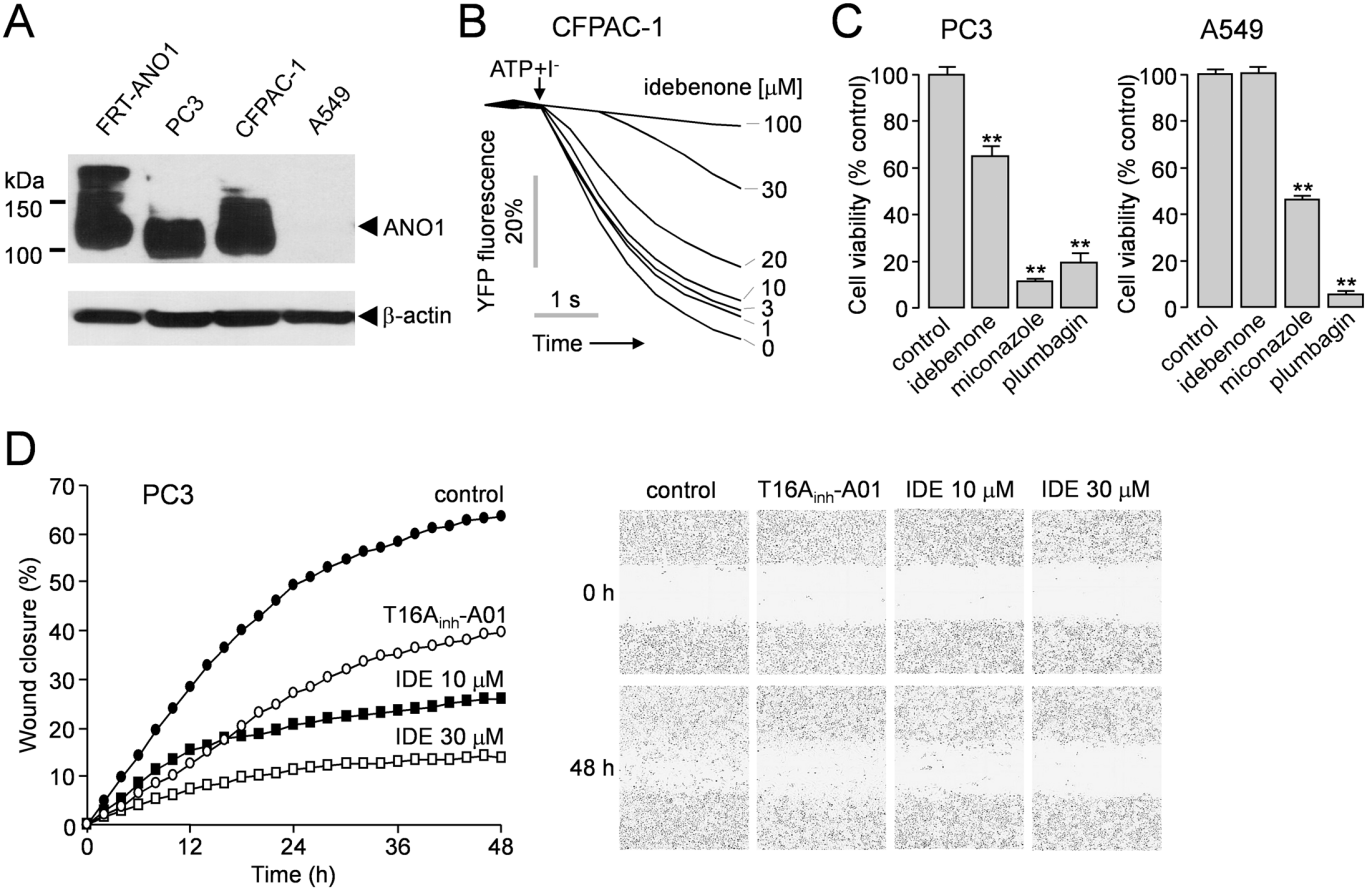
ANO1 expression in adenocarcinoma cell lines and effect of ANO1 inhibitors on the cell viability and migration. (A) Immunoblot of ANO1 protein in FRT-ANO1, PC3, CFPAC-1 and A549 cells. Representatives of three sets of studies are shown. (B) Effect of idebenone on CaCCs was measured in CFPAC-1 cells expressing halide sensitive mutant YFP. CaCCs were activated by 100 μM ATP. (C) PC3 and A549 cells were treated with idebenone (30 μM), miconazole (30 μM) and plumbagin (30 μM), and cell proliferation was measured after 2 days using MTS assays (mean ± S.E., n = 6). (D) Wound healing assay in PC3 cells. The cells were treated with T16A_inh_-A01 (30 μM) and idebenone. (left) The wound closure was quantified at every 2 h post-wound (mean ± S.E., n = 5). (right) Representative images taken at 0 h and 48 h post wounding (× 10). **P < 0.01, Students’ unpaired t-test.

Three types of human adenocarcinoma cell lines were tested to determine the effect of idebenone on the endogenous CaCCs activity and cell growth and migration. Western blotting revealed that endogenous ANO1 is expressed at high levels in PC3 and CFPAC-1 (human pancreatic ductal adenocarcinoma derived) cells but not in A549 (human lung adenocarcinoma derived) cells (Fig 4A). The YFP fluorescence quenching assay with CFPAC-1 cells expressing the halide sensing mutant YFP revealed that idebenone potently blocked ATP-induced CaCC chloride channel activation in a dose-dependent manner (Fig 4B). To investigate whether the ANO1 inhibitors affect the cell growth, idebenone, miconazole and plumbagin were applied to PC3 cells expressing high levels of ANO1 and A549 cells not expressing ANO1. The results are shown in Fig 4C, and they reveal that idebenone significantly inhibited the cell growth in PC3 cells but not in A549 cells. Miconazole and plumbagin showed strong inhibition of cell growth in both PC3 and A549 cells, but it was not surprising because in several previous investigations, it was shown that miconazole and plumbagin inhibited cell growth of various human cancer cells through different mechanisms [24–29]. The effect of ANO1 inhibition by idebenone on cell migration was determined using a wound healing assay in PC3 cells. In the assay, control PC3 cells covered 63.6 ± 2.5% (n = 5) of the wound, whereas T16A_inh_-A01 (30 μM) and idebenone (10 and 30 μM) treated cells covered 39.6± 2.5, 26.1 ± 1.8 and 13.8 ± 0.8% (n = 5) of the wound at 48h post-wound, respectively (Fig 4D).

**Fig 5:**
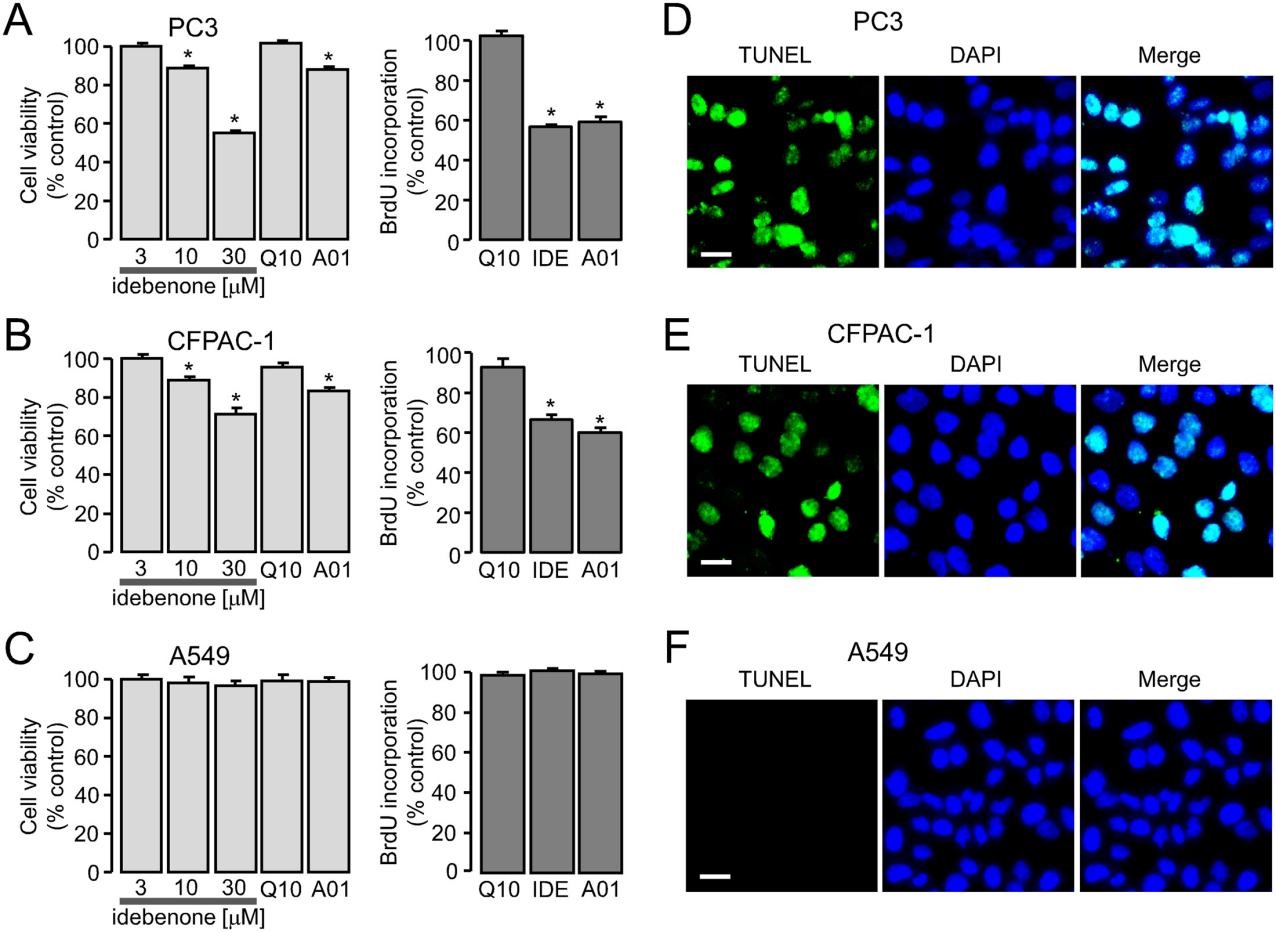
Effects of ANO1 inhibition on the cell proliferation and apoptosis in PC3, CFPAC-1 and A549 cells. (A-C) PC3, CFPAC-1 and A549 cells were seeded in 96 well plates, and after 24 h incubation, they were treated with the indicated concentrations of idebenone (IDE), 100 μM coenzyme Q10 (Q10) and 10 μM T16A_inh_-A01 (A01) in MTS assay. IDE (30 μM), coenzyme Q10 (100 μM) and T16A_inh_-A01 (10 μM) were applied to the cells in BrdU assay. Cell proliferation was estimated after 2 days via MTS (left) or BrdU (right) assay (mean ± S.E., n = 6). (D-F) PC3, CFPAC-1 and A549 cells were treated with 30 μM idebenone. The cells were stained with TUNEL (terminal deoxynucleotide transferase-mediated dUTP nick-end labeling, green) and DAPI (4,6-diamidino-2-phenylindole, blue). Scale bars represent 20 μm. *P < 0.05, Students’ unpaired t-test.

To further determine the effect of idebenone on cell proliferation, we observed cell proliferation in response to idebenone using MTS assay and BrdU assay in PC3, CFPAC-1 and A549 cells (Fig 5). In this study, coenzyme Q10 was used as negative control because it does not inhibit ANO1/CaCCs even though idebenone is a synthetic analog of coenzyme Q10 (Fig 2F). The cells were treated with idebenone (3, 10 and 30 μM), coenzyme Q10 (100 μM) and T16A_inh_-A01 (10 μM), and the cell proliferation was quantitatively estimated after 2 days. In PC3 and CFPAC-1 cells, idebenone inhibited cell viability and BrdU incorporation in a dose-dependent manner, but idebenone and T16A_inh_-A01did not affect cell viability and BrdU incorporation in A549 cells (Fig 5A–5C). Coenzyme Q10 did not block the cell proliferation in all the cell lines. Downregulation of ANO1 induces apoptosis in several cancer cell lines overexpressing ANO1 [14, 16, 30]. To investigate whether idebenone induces apoptosis in ANO1 expressing cells, TUNEL staining was performed in the adenocarcinoma cell lines. PC3, CFPAC-1 and A549 cells were incubated with 30 μM idebenone for 48 h, and then DNA damage was monitored using the TUNEL assay. TUNEL positive cells were detected in PC3 and CFPAC-1 cells but not in A549 cells (Fig 5D–5F).

## Discussion

ANO1, a recently identified CaCC, is strongly overexpressed in various tumor types including HNSCC, GIST, breast and prostate cancer. Notably, Recent evidence strongly suggests ANO1 involvement in cell proliferation, cell migration, tumorigenesis and cancer progression [12, 13, 16, 31]. Several studies have indicated that ANO1 may be used as a therapeutic target of tumors because down regulation of ANO1 showed potential therapeutic benefits on HNSCC, ESCC, GIST, breast and prostate cancer [14, 16, 30, 32, 33]. Thanks to recent identification of ANO1, few small molecule ANO1 inhibitors are available such as CaCC_inh_-A01, tannic acid, T16A_inh_-A01, and MONNA [17, 19–21], but the pharmacological property and the mechanisms of action of the inhibitors are still unclear. In this study, we performed a cell-based screening to identify novel ANO1 inhibitors with a collection of drug like compounds and natural products. Interestingly, we discovered that idebenone strongly inhibited ANO1.

Idebenone [2,3-dimethoxy-5-methyl-6-(10-hydroxydecyl)-21,4-benzoquinone] is a synthetic short chain benzoquinone as an analogue of ubiquinone (Coenzyme Q10). Idebenone is currently being investigated for the treatment of a number of mitochondrial and neuromuscular diseases, such as Friedreich’s ataxia (FRDA), mitochondrial encephalopathy with lactic acidosis and stroke-like episode (MELAS) and Duchenne muscular dystrophy (DMD), due to its ability to serve as an electron carrier in the mitochondrial electron transport chain and its powerful antioxidant activity. Beneficial clinical effects of idebenone have been reported in the idebenone treated patients with FRDA (300–2250 mg/day; 0.5–5 years), MELAS (90–270 mg/day; 163 days) and DMD (450 mg/day; 12 months) [34–36]. In addition, results of large clinical trials showed that idebenone was safe and well tolerated [37, 38].

We showed that idebenone potently inhibited the ANO1 activity in YFP fluorescence quenching assay (Fig 1C) and electrophysiological studies (Figs 2A and 3A). To find out whether antioxidant and electron carrier activity of idebenone affect ANO1 channel function, we observed the effect of CoQ10 on ANO1 channel activity because idebenone and CoQ10 share the same substitution pattern of the quinone moiety and differ only in the alkyl tail attached to the C6-carbon atom of their quinone ring. As shown in Fig 2F, pretreatment of 100 μM CoQ10 slightly (but not significantly) increased ANO1 currents instead of inhibiting ATP-induced ANO1 currents in ANO1 expressing FRT cells. In addition, CoQ10 did not block the cell viability and proliferation in several ANO1 expressing cell lines (Fig 5), and idebenone did not affect CFTR and intracellular calcium signaling (Fig 2C and 2D). Together, the above results suggest that the mechanism of action of idebenone on ANO1 inhibition may be direct rather than through its antioxidant and electron carrier ability. There is still a possibility that idebenone affect cell proliferation and apoptosis via the other pathways. However, idebenone showed potent inhibition of ANO1 and cell proliferation in ANO1 expressing cells, and an ANO1 specific inhibitor T16A_inh_-A01 showed similar response on cell proliferation (Fig 5). This result suggests that idebenone induced ANO1 inhibition is at least partially involved in the cytotoxic effect of idebenone. In Fig 4D, we showed that strong inhibition of cell migration into a wound area by idebenone. In wound healing assay, we used serum-free cell culture media to reduce the cell growth because both cell proliferation and migration affect the rate of wound healing. Serum deprivation resulted in ~54% growth inhibition (data not shown) and the inhibitory effect of idebenone on wound healing was stronger than that of idebenone on cell proliferation (Figs 4D and 5A). These results suggest that idebenone inhibits cell migration even though growth inhibition by idebenone can affect the result.

In clinical trial, patients have been treated with idebenone up to 2,250 mg/day. When healthy male subjects received a single oral dose of 750 mg of idebenone three times per day, the maximum plasma concentrations (C_max_) of parental and total idebenone were 22.4 and 8,158 ng/mL, respectably [39]. The very low plasma concentrations of parent idebenone (~66 nM) due to rapid metabolism and the high plasma concentrations of total idebenone (~24 μM) including idebenone and its metabolites suggest that further studies on the effect of idebenone metabolites on ANO1 activity are needed to evaluate the role of ANO1 inhibition in the various biological effects of idebenone.

In this study, we also showed that miconazole and plumbagin potently inhibited ANO1 channel activity without altering the intracellular calcium concentration in FRT and HT-29 cells (Figs 1C and 2C). These two compounds strongly decreased cell growth of PC3 cells expressing high levels of ANO1 at 30 μM concentration showing near complete inhibition of ANO1 (Fig 4C). Miconazole, an imidazole antifungal agent, inhibits ergosterol biosynthesis and is commonly applied topically to treat superficial mycoses [40]. Recent studies have shown that miconazole inhibits cell growth by modulation of p53-associated signaling pathway and suppression of HIF-1α protein synthesis, but the underlying mechanism is still not well understood [24, 25]. Plumbagin, a quinonoid obtained from the roots of Plumbago zeylanica, has been found to possess highly potent biological activities including anticancer properties. Plumbagin reportedly has chemopreventive properties against several cancer cells such as inhibition of cell growth in breast cancer cells, inducing apoptosis in pancreatic cancer cells, and inhibition of invasion and migration in liver cancer cells via various pathways including inhibition of AKT and downstream targets, but the underlying molecular mechanism is still unclear. [26–29]. In previous studies, miconazole and plumbagin showed dose-dependent inhibition of cell growth in human cancer cells with IC_50_ in the range 10–20 μM and 3–10 μM, respectively [24, 26–28]. In Fig 1C, we showed that 10–20 μM miconazole and 3–10 μM plumbagin can significantly inhibit ANO1 activity. Thus, ANO1 inhibition by miconazole and plumbagin may be one of possible mechanisms of cytotoxic effects in cancer cells. To the best of our knowledge, this is the first work that shows idebenone, miconazole and plumbagin inhibit ANO1 chloride channel, and inhibition of ANO1 channel activity by these compounds may be, at least partially, involved in inhibition of cell proliferation.

In Fig 4A, ANO1 expression was analyzed by Western blotting, and it is revealed that the size and thickness of ANO1 bands were different in FRT-ANO1, PC3, CFPAC-1 cells. In the present study, we did not clarify the difference of ANO1 bands in the three cell lines. The alternative splicing of ANO1 [6], different ANO1 expression levels, and post-translational modifications such as glycosylation may create different sized ANO1 proteins.

In conclusion, this study shows that idebenone is a novel potent inhibitor of ANO1, and it did not affect intracellular calcium signaling and CFTR chloride channel activity. Idebenone potently inhibited ANO1/CaCC channel activity in PC-3 and CFPAC-1 cells, and significantly reduced cell proliferation, inhibited cell migration and induced apoptosis in these cells. These data suggest that idebenone may be a powerful candidate in the development of novel therapeutic agents for the prevention and treatment of cancer.
